# A physiotherapist-led biopsychosocial education and exercise programme for patients with chronic low back pain in Ghana: a mixed-methods feasibility study

**DOI:** 10.1186/s12891-024-08118-1

**Published:** 2024-12-18

**Authors:** Paapa Kwesi Ampiah, Paul Hendrick, Fiona Moffatt, Josephine Ahenkorah Ampiah

**Affiliations:** 1 Division of Physiotherapy, Department of Health Sciences, College of Health, Medicine and Life Sciences, Brunel University of London, London, UK; 2 Division of Physiotherapy and Rehabilitation Sciences, School of Health Sciences, University of Nottingham, Nottingham, UK; 3 Institute of Health and Social Care, Division of Physiotherapy, Sports Rehabilitation and Chiropractic, London South Bank University, London, UK

**Keywords:** Physiotherapy, Biopsychosocial, Exercise, Patient education, Feasibility, Mixed-methods

## Abstract

**Background:**

Low back pain is a common musculoskeletal condition which causes substantial disability globally. The biopsychosocial model of management has been recommended in national and international guidelines for the management of patients with chronic low back pain (CLBP). However, biopsychosocial approaches are predominantly delivered in high income countries (HICs), although the prevalence of LBP is substantially higher in low- and middle-income countries (LMICs) especially in Africa (39%; 95% CI 30–47). Understanding the effectiveness of BPS interventions in LMICs especially in Africa is underexplored, with substantial inequity between research from HICs and LMICs. Ghana is a LMIC where the effectiveness of biopsychosocial interventions has been underexplored. Therefore, the aim of this study was to explore the feasibility of delivering a physiotherapist-led BPS programme for the management of patients with CLBP in Ghana.

**Methods:**

This was a mixed-methods, sequential, pretest-posttest feasibility study. Participants involved thirty patients with CLBP. The biopsychosocial intervention involved an exercise and patient education programme based on principles of cognitive behavioural strategies with emphasis on self-management. The biopsychosocial intervention was delivered for six weeks for each participant. Feasibility outcomes regarding management and processes were captured pre-intervention, post-intervention, and three-months post intervention. Semi-structured interviews were conducted post-intervention to explore participants’ experiences with the biopsychosocial intervention. Patients’ demographics were collected at baseline. Patient reported outcome measures such as intensity of pain, disability, pain catastrophising, kinesiophobia, self-efficacy, and general quality of life, were collected pre-intervention, post-intervention and at three-months follow-up. Qualitative analysis explored participants’ experiences regarding the acceptability of the biopsychosocial intervention.

**Results:**

The results of this feasibility study demonstrated that the training programme was acceptable to physiotherapists. Recruitment rate (5 patient participants per week − 100% recruitment met), retention rate post-intervention (90%), data completion rate post-intervention (99.8%) and intervention fidelity (83.1%), all met feasibility thresholds. There were no adverse events. Qualitative data also demonstrated that the biopsychosocial intervention was acceptable to participants.

**Conclusion:**

This study has established the potential to deliver a biopsychosocial intervention programme in a Ghanaian hospital setting. This biopsychosocial intervention therefore shows promise, and the result of the study provides a platform to develop future clinical studies.

**Supplementary Information:**

The online version contains supplementary material available at 10.1186/s12891-024-08118-1.

## Introduction

Low back pain (LBP) is a musculoskeletal condition which is common and experienced by many people [1]. LBP is also the principal cause of years lived with disability (YDL) [1, 2], and affects people from both high-income countries (HICs) and low- and middle-income countries (LMICs) [3]. Globally, LBP is highly prevalent and has seen a substantial increase in YLD of 9.4% between 1990 and 2010 (1549–1694, per 100,000 people) [4]. The trends in prevalence are substantial in LMICs particularly in Africa (39% - point prevalence; 57% annual prevalence); attributable to increasing age and a high level of manual duties people engage in, although LBP receives minimal prioritization compared to other health conditions [5].

The main cause of LBP is unknown/non-specific, accounting for about 90% of all LBP [6, 7]. At the acute stage, the prognosis is typically favourable, especially when the LBP is non-specific [8]. However, when patients transit into chronicity, the prognosis is usually poor [9]. This state is aligned with patients’ physical, and psychosocial factors [8, 9]; hence, it is important to explore these factors during management of chronic low back pain (CLBP). CLBP management is aimed at reducing the physical and psychosocial factors within a biopsychosocial (BPS) model [8].

The BPS model is recommended by global guidelines like the National Institute of Care Excellence (NICE) [10]. Evidence suggests that making positive modifications in physical and psychosocial factors (for example, self-efficacy, catastrophising), mediate and predict favourable outcomes for patients with CLBP [11–13]. There are varied conservative evidence-based BPS interventions that are applied for the management of CLBP; including, cognitive functional therapy (CFT) [14], exercise-informed behavioural graded activity [15, 16], physical activity-informed cognitive behavioural therapy [17, 18], and patient education plus exercise approaches [19, 20]. These BPS interventions are mostly applied in HICs [21, 22]. CFT works on the principle of challenging unhelpful behaviours (for example, deconditioning and pain behaviours, muscle guarding) and reversing negative beliefs and cognitive factors (for example, hypervigilance, catastrophising), in a functionally concise, progressive, and cognitively integrated manner [14]. Similarly, a reversal of cognitive factors, maladaptive beliefs and behaviours, with emphasis on self-management underpin exercise informed behavioural graded activity [15, 16], physical activity-informed cognitive behavioural therapy [17, 18] and patient education plus exercise approaches [19, 20].

It is important to note that BPS factors are not peculiar to HICs [23]; the BPS model is also recommended by the Global Spine Care Initiative for managing patients with CLBP in LMICs [24]. However, the Global Spine Care Initiative recognizes that health systems of LMICs are low-resourced, and recommend interventions involving physical activity/exercise, self-management, and advice/education within a BPS model [24]. Despite these recommendations [24], there is limited exploration or delivery of BPS approaches in LMICs [21, 22]. This evidence has been reinforced by a systematic review revealing a paucity of high-quality BPS informed physiotherapist-led studies, for managing patients with CLBP, in LMICs [23]. Evidence from Ghana, a LMIC, also suggests a biomedical approach (for example, x-ray imaging, bed rest on a firm mattress) to managing CLBP [25]. The Medical Research Council (MRC) advocates that in the development/delivery of complex interventions, researchers need to assess the feasibility of delivery [26]; therefore, it was essential to investigate the feasibility of delivering BPS approaches in resource-limited LMICs like Ghana. The aim of this study was to assess the feasibility of delivering a physiotherapist-led BPS management programme for patients with CLBP in Ghana.

The specific objectives were to establish:

Quantitatively, whether it was feasible to recruit and retain participants, capture data, assess the treatment compliance, and fidelity of the BPS intervention.

Qualitatively, whether it was feasible to train physiotherapists to deliver the BPS intervention, whether there were any adverse effects, and whether the intervention was acceptable to patient participants.

The research question was: What is the feasibility of delivering a physiotherapist-led BPS exercise and patient-education programme for patients with CLBP in Ghana? **Method**.

## Design

This was a mixed-methods, sequential, pretest-posttest quasi-experimental, feasibility study. The rationale for applying a quasi-experimental design was because it addressed the research question/aim. Furthermore, there is limited evidence demonstrating the feasibility for conducting high quality randomised controlled trials (RCTs) within the context [23]; thereby limiting the ability to plan/conduct high-quality RCTs. There was an initial training programme for physiotherapists, followed by the feasibility study, and qualitative interviews. The complete protocol for this study has been published previously [27]. The study was conducted in an out-patients physiotherapy department within one of Ghana’s major teaching hospitals, Komfo Anokye Teaching Hospital (KATH). The TREND statement guided reporting [28].

### Participants and therapists

Physiotherapists and patients with CLBP were recruited for this study (Supplement 1 - eligibility criteria). The screening questionnaire for eligibility was administered by the principal investigator (PI). A sample of two physiotherapists were recruited. Eight physiotherapists (*n* = 8) managed patients with LBP at the time of recruitment; however, majority were time constrained and could not volunteer. In the circumstances, two physiotherapists were deemed adequate and aligned with the a priori feasibility criterion. Identification/recruitment of physiotherapists was facilitated by the head of physiotherapy at KATH who was the gatekeeper. The physiotherapists were both male, aged 27 and 34, with 2- and 7-years’ working experience.

**Fig. 1:**
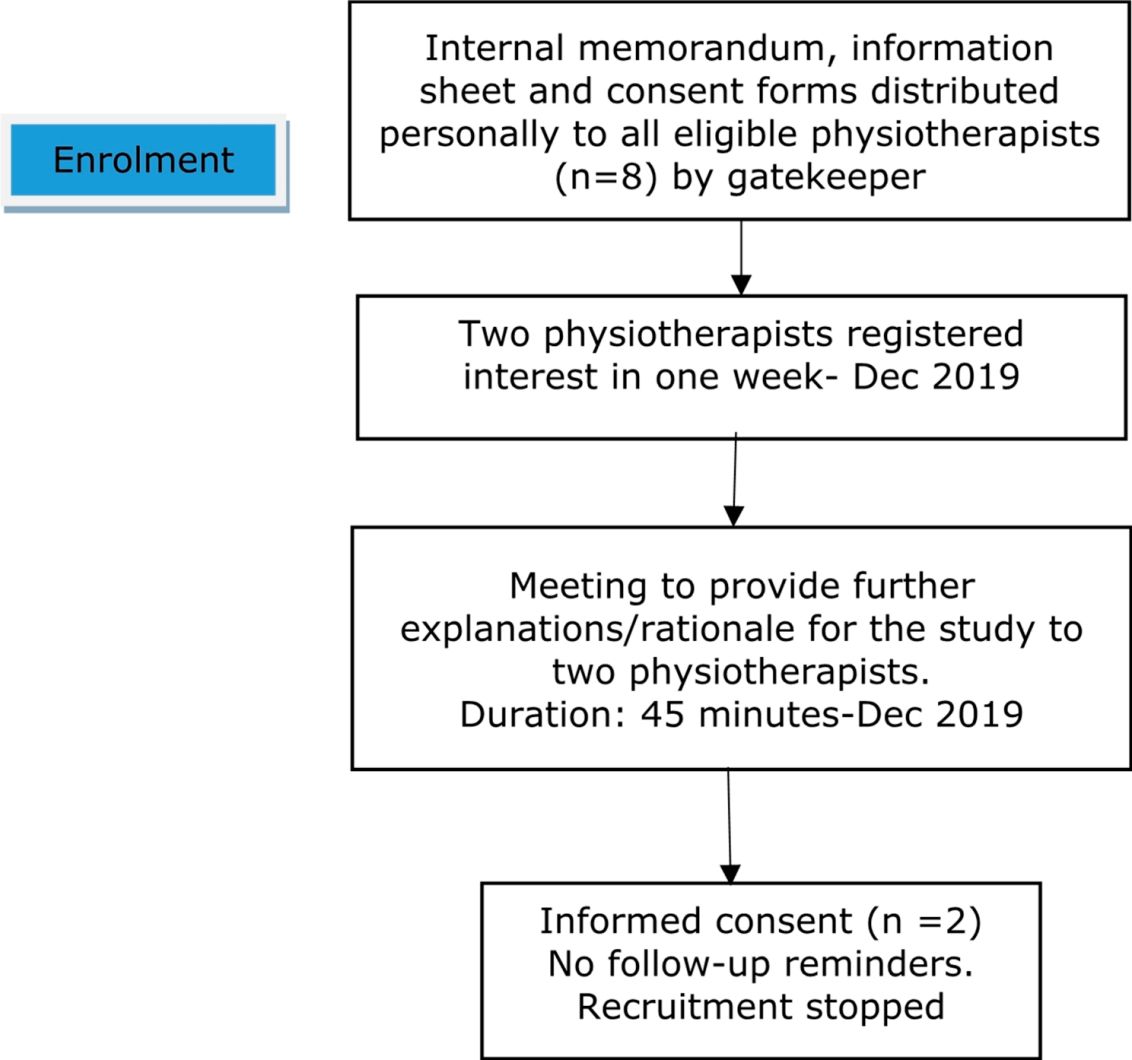
Recruitment process for physiotherapists

**Fig. 2:**
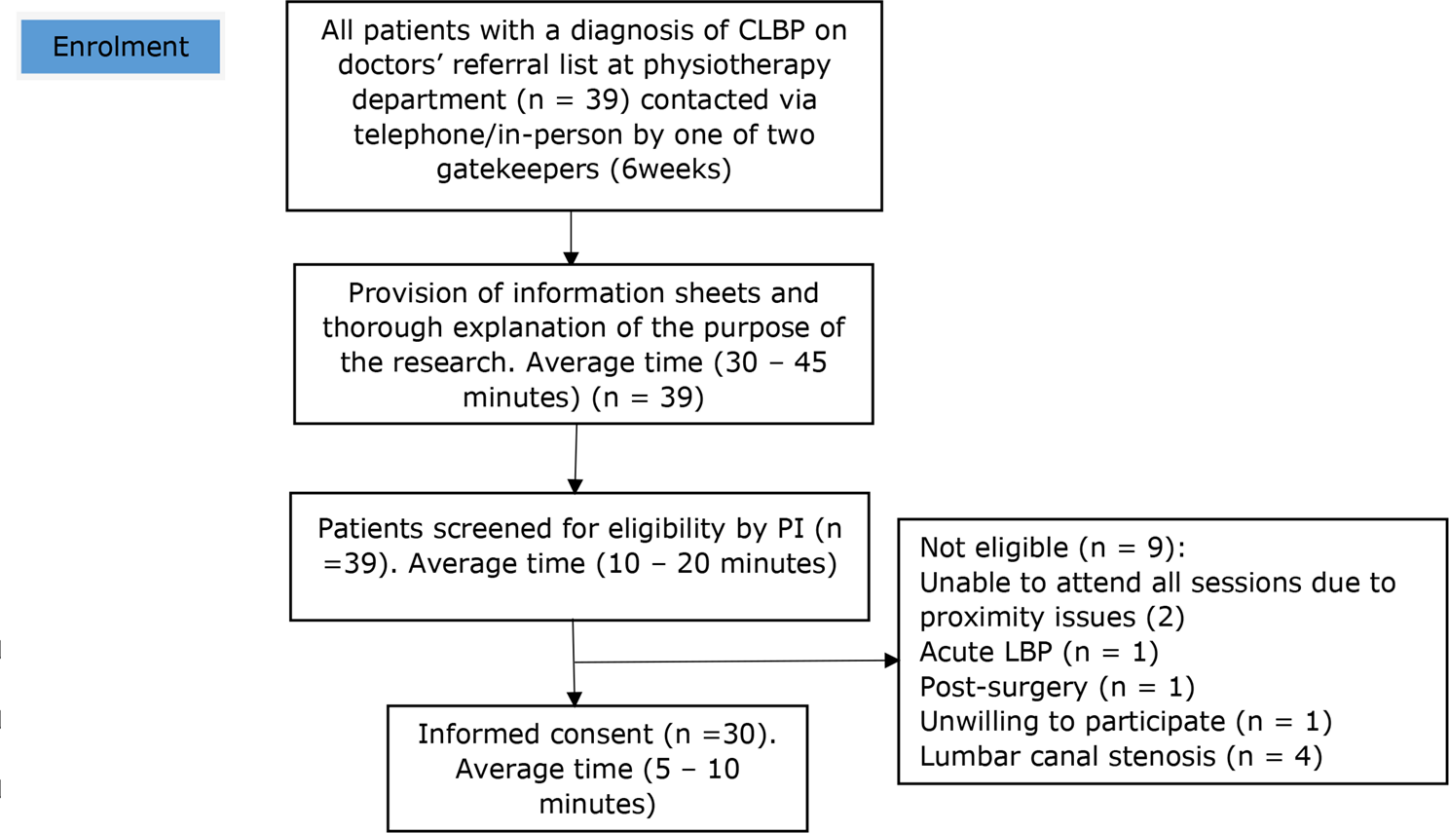
Recruitment process for patients

Patients were recruited from the doctors’ referral list at the Physiotherapy Department of KATH. All patients with a diagnosis of CLBP, on the doctors’ referral list between December 2019 to mid- January 2020, were eligible for a telephone call or an initial in-person explanation of the study aims/considerations by the clinical gatekeeper. This was followed by screening of interested participants by the PI. Figures 1 and 2 demonstrate flowcharts of the recruitment processes for physiotherapist and patient participants, respectively. Patient participants were non-randomly allocated to the physiotherapist participants (PT1 and PT2) by the PI.

Once patients completed the eligibility screening and consented to participate in the research, they were allocated to a physiotherapist participant. Allocation was based on the availability of the physiotherapist participants. Due to the nature of feasibility studies, a formal sample size was not calculated [30]. A sample of thirty patients with CLBP was anticipated to be recruited. This number was deemed adequate based on previous feasibility studies [31], and aligned with the average number of new patients with LBP seen in the physiotherapy department of KATH monthly. The qualitative aspect involved interviews for the two physiotherapists and six patient participants who consented to participate in the interviews. Six patient participants were deemed adequate based on data saturation and aligned with the a priori feasibility criterion.

## Interventions

### Training programme

**Fig. 3:**
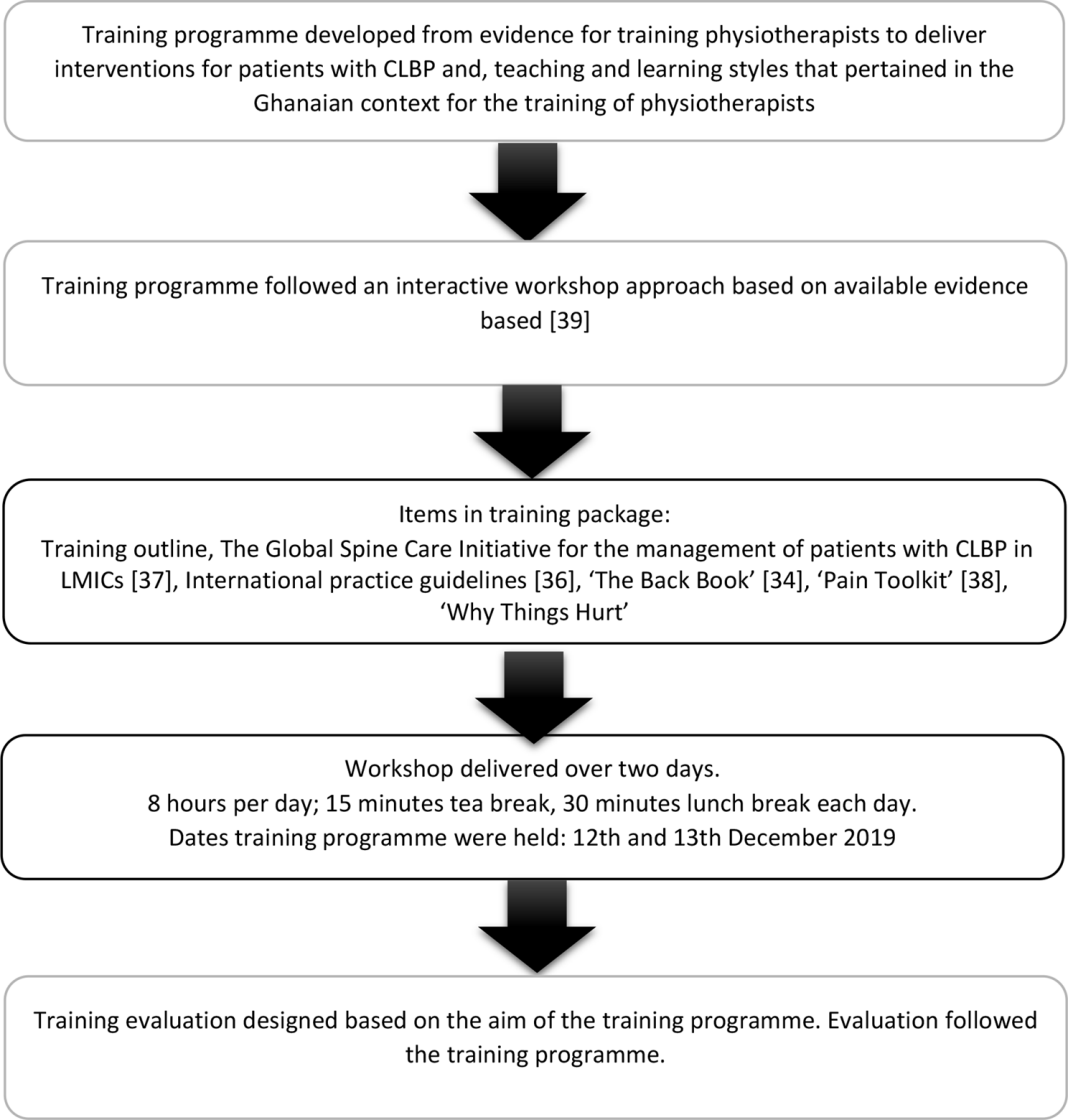
Processes in involved in the development, delivery and evaluation of the training programme

The training programme was structured based on the content of the BPS intervention, within an eight-hour period. The training for physiotherapist participants was held over two days (12th and 13th December 2019). Physiotherapists’ experiences with the training programme were assessed using a training evaluation form. The PI delivered the training programme while a voluntary research assistant prepared the training room and distributed the training package. Supplement 2 presents an outline of the training programme. Figure 3 illustrates the training processes.

## Biopsychosocial intervention

The research team considered the evidence-base for developing interventions to inform the development of the BPS intervention (Supplement 3). The taxonomy of approaches to intervention development, which recommends combining published research and formal theories, was applied in developing the BPS intervention [38]. The BPS intervention applied combined exercise (motor control exercises, stretching exercises and aerobic exercises) and patient education based on principles of cognitive behavioural strategies emphasizing self-management, reshaping LBP beliefs and education on the influence of maladaptive beliefs on LBP [27]. The exercise component lasted between forty-five minutes to one hour. The patient education component lasted between forty-five minutes to one hour and preceded the exercise. Participant physiotherapists delivered the exercise component twice a week for six weeks, whilst the education component was delivered once a week for six weeks. Both components were delivered on an individual basis [27]. All patient participants were advised (by participant physiotherapists) to achieve moderate intensity physical activity at home (at least 150 min every week) through daily aerobic activity (moderate intensity walking for 30 min five times a week) [39, 40]. Patient participants were also given ‘The Back Book’ [33]. Supplement 4 summarises the intervention and how it fulfils the definition of a BPS intervention.

## Outcome measures

Data collection spanned 6 months (December 2019 - June 2020). An open-ended evaluation form assessing the acceptability of the training programme was completed by participating physiotherapists. Feasibility outcomes (primary outcomes) for participants that were tested included recruitment and retention rate, treatment fidelity, dropout rate, treatment compliance, data completion rate, and adverse events [20, 42, 43]. The a priori feasibility thresholds for the primary outcomes were as follows; recruitment rate - ≥ 3 patient participants recruited per week; treatment fidelity − 80% ≥ attainment of intervention fidelity; and drop-out rate - ≥ 20% dropout of patient participants. Supplement 5 presents the operational definitions and a priori criteria for all the feasibility outcomes. All feasibility outcomes were collected by the PI or voluntary research assistant. Secondary outcome measures (patient reported outcome measures) were collected at baseline, post intervention, and 3 months (Supplement 6). Patient reported outcome measures applied in this study included numeric rating scale for pain, Roland Morris disability questionnaire for disability, Generic health outcome Euro-QOL (EQ-5D-5 L) for general quality of life, pain catastrophising scale for pain catastrophising, general self-efficacy scale for self-efficacy, and Tampa scale of kinesiophobia for kinesiophobia.

**Fig. 4:**
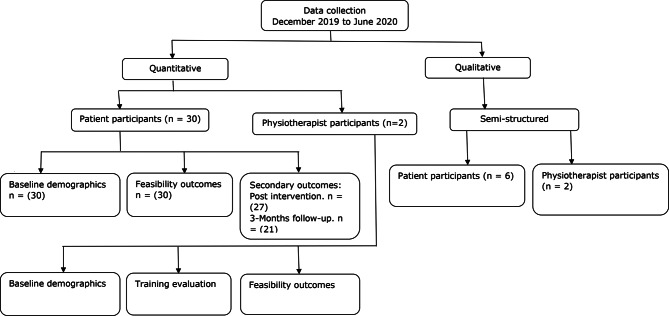
Data collection processes in this study

Semi-structured interviews were used to collect qualitative data from participants and ensured interview flexibility [44]. The interview guide was informed from previous feasibility studies [45], and the objectives of the study. All interviews were audio recorded. A research diary was used to capture reflexive observations/interview notes. The qualitative data collection spanned six weeks (February - March 2020). The data collection processes for qualitative and quantitative data are illustrated in Fig. 4. Qualitative analysis explored participants’ experiences regarding the acceptability of the intervention. All interviews were conducted by the PI.

### Data analysis

Quantitative data was managed and analysed using Microsoft Office Excel and SPSS Version 24. Qualitative data was managed using Microsoft Office Word. Participants’ demographics and baseline characteristics were summarised descriptively using means, standards deviation, percentages, medians and interquartile ranges. Since the study was a feasibility study, accounting for missing data and intention-to-treat analysis was not conducted [45]. Analysis of feasibility thresholds were conducted based on the a priori feasibility criteria [27]. Qualitative data derived from participant interviews was analysed thematically as described by Braun and Clarke [64]. Physiotherapists’ evaluation of the training programme was assessed as either negative or positive feedback with exemplar texts. A complete description of the data analysis has been previously published [27].

## Results

### Patient demographic characteristics

We successfully recruited thirty (*n* = 30) patient participants. Of the 30 patient participants recruited, 80% were female (*n* = 24). The duration of LBP ranged from 3 months − 120 months. Missing data was managed by identifying any baseline/outcome data that were not entered by the patient participants. Table 1 summarizes the demographics characteristics of participants.

**Table 1:** Baseline demographics and self-reported measures (*n* = 30)

Variable	Data; mean (SD)
Age	48.6 (13.5)
Age range	20–71
Gender**	Male 6 (20%) Female 24 (80%)
Religion**	Christian 28 (93.3%) Muslim 2 (6.7%)
Duration of low back pain (months)*	12 (4.75–30)
Previous LBP **	Yes 27 (90%) No 3 (10%)
Level of education **	Primary 4 (13.3%) Junior high school 8 (26.6%) Senior high school 6 (20.0%) Tertiary 7 (23.3%) No formal education 2 (6.7%) Training college 3 (10.0%)
Employment status **	Employed 9 (30%) Unemployed 11 (36.7%) Self-employed 10 (33.3%)
Marital status **	Married 17 (58.6%) Single 4 (13.8%) Divorced 3 (10.3%) Widowed 5 (17.2%)
NRS	7.4 (1.16)
RMDQ	15.9 (3.89)
EQ-5D-5 L	3.5 (0.48)
Health VAS	44.0 (10.70)
PCS	37.6 (7.88)
GSES	14.9 (2.76)
TSK	53.3 (5.66)

*Median (interquartile range), **Number (Percentage), NRS; Numeric Rating Scale, RMDQ; Roland Morris Disability Questionnaire, EQ-5D-5 L; EuroQol 5 dimensions, VAS; Visual Analogue Scale, PCS; Pain Catastrophising Scale, GSES; General Self Efficacy Scale, TSK; Tampa Scale of Kinesiophobia

### Feasibility outcomes

#### Recruitment

**Fig. 5:**
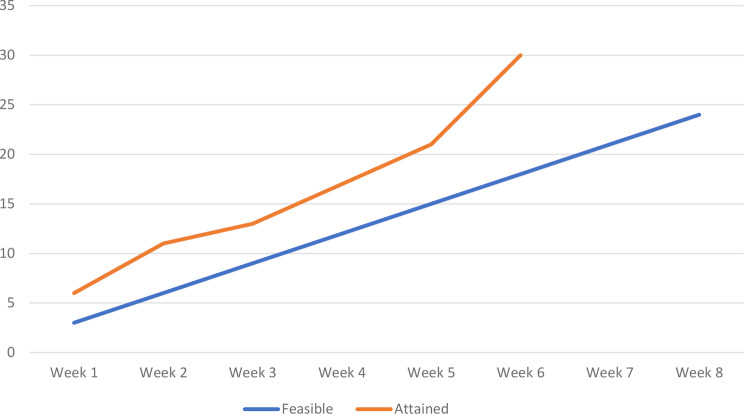
Figure showing feasibility criteria attained for recruitment of patients

**Fig. 6:**
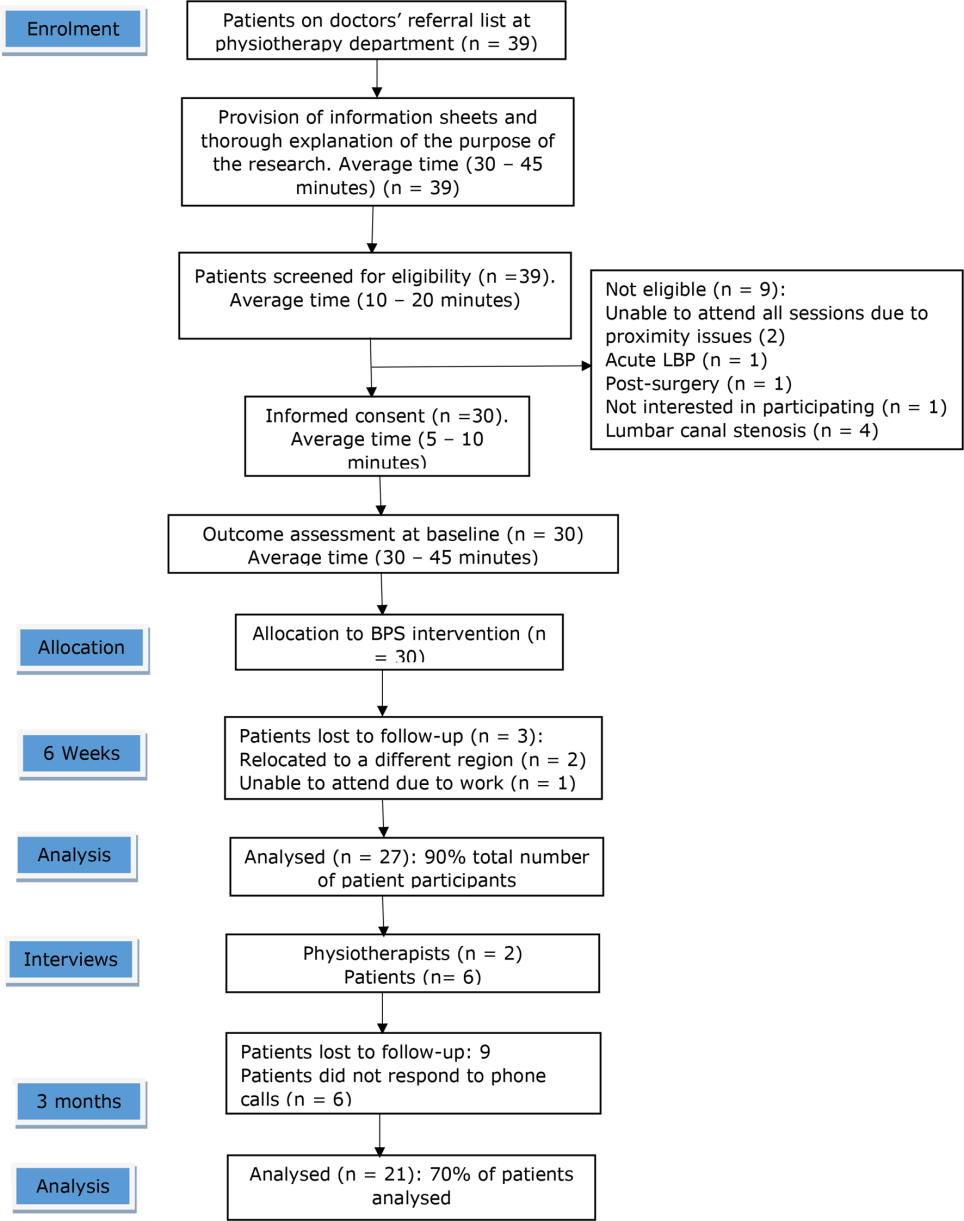
Flow chart representing course of participants through the study

A mean of 5 patients were recruited per week (Fig. 5), spanning 6 weeks, denoting that the a priori feasibility criterion (3 ≥ patients recruited per week) was achieved. Thirty-nine (*n* = 39) potential patient participants were screened for potential recruitment. There were no concerns raised by the potential patient participants during the process of screening for eligibility. Consent rate was calculated as a percentage of patient participants who consented to participate against those who met the inclusion criteria. Consent rate was 100%. Two physiotherapist participants were recruited for this study, denoting that the a priori feasibility criterion (2 ≥ physiotherapists recruited) was achieved. Figure 6 illustrates the flow of physiotherapist and patient participants.

### Training programme

The results showed that the training programme was acceptable. Physiotherapist participants commented that the training setting and facilities were adequate.

*“The setting was well-known and friendly environment*,* devoid of tension. A well organised lab session*,* where exercise demonstration and practice was feasible” PT2*.

Physiotherapist participants further suggested that the training objectives were met.

*“The delivery was clear*,* and the handouts too further explained what was delivered” PT1*.

Physiotherapist participants opined that the session and discussions were useful. Regarding aspects of the training programme that could be improved, there was no consensus. One physiotherapist found every aspect useful. The other physiotherapist suggested that it would have been useful to add other LBP classifications in the delivery of the training programme in addition to NSCLBP. However, that would have gone beyond the scope of this study.

### Patient participant allocation

Following the recruitment and training programme, patient participants were allocated to either PT1 or PT2. Overall, the results showed that all eligible patient participants were allocated to the participating physiotherapists by the PI (PT1 = 12; PT2 = 18).

### Rate of data completion

Pre-intervention/baseline data completion was 99.2%, post intervention data completion was 99.8%, and three-month follow-up data completion was 99.7%. Each data completion rate had less than 1% missing data. Feasibility thresholds were therefore met at all three assessment timepoints.

### Dropout rate

Post-intervention dropout rate was 10%, denoting a patient retention rate of 90%. Three patient participants dropped out after the first (*n* = 1 patient) and second (*n* = 2 patients) week of the BPS intervention programme. A further six (*n* = 6) patient participants dropped out at three months. This resulted in a dropout rate of 30%, which meant the feasibility threshold at three-months follow-up was not achieved. However, it is important to note that there was a change in the mode with which patient participants completed the outcome measures at three months follow-up, which was different from the initially agreed mode. The change was due to the inception of COVID-19, which meant participants could not present the completed outcome measures in person; the research team telephoned each patient participant. Six patient participants did not respond after two attempts within a timeframe of two weeks. An amended ethics approval was acquired before carrying out the change. It is important to note that the patients that dropped out did not present any specific characteristics of interest; therefore, the conclusion was that COVID-19 potentially accounted for this.

### Adverse events

No adverse events were recorded, although two (*n* = 2) patient participants had to reschedule their management sessions due to other underlying medical conditions unrelated to their CLBP.

### Patient participants’ compliance with management schedules

Compliance with management schedules by patient was assessed with adherence to out-patient sessions, and adherence to the recommended home exercises. The results from the out-patient sessions showed an adherence rate of 80.56%. The majority (*n* = 20) of patient participants completed at least nine out of the twelve scheduled sessions. A summary of the patient participants’ reasons for non-compliance included time constraints due to work and household (family) commitments. Overall, the feasibility threshold was achieved. Regarding compliance with home exercises, only five (*n* = 5; 18.5%) patient participants returned their exercise diaries to the participating physiotherapists. A summary of the reasons given for non-compliance by the remaining patient participants included constraints due to work and household demands.

### Fidelity of the BPS intervention

Intervention fidelity was assessed based on the National Institute of Health Behaviour Change Consortium (NIHBCC) [65] checklist. Assessment of the fidelity component on training of providers showed that six out of the seven components were achieved. Overall, 83.1% fidelity of the BPS intervention was achieved, meaning the feasibility threshold was achieved. Table 2 illustrates the results of fidelity testing. Furthermore, Table 3 presents all the results of the feasibility outcomes.

**Table 2:** Outcome of monitoring and assessment of intervention fidelity

Indicator/ Operational Definition	Component of fidelity		Assessment						Proposed Assessment in clinical study
			1st	2nd	3rd	4th	5th	6th NA	In addition to review by expert panel/protocol review committees, the design of the study could benefit from involving patients and physiotherapists (e.g., using PPIs or community participatory research [65]
**Study design**: Involved the processes of the BPS intervention design, (including required patient and HCP participants) based on a biopsychosocial approach to care within a Ghanaian context.	1	Provide information about treatment dose in the intervention condition: Length of contact (minutes) Number of contacts Content of treatment Duration of contact over time	1	NA	NA	NA	NA		
	2	Provided information about treatment dose in the comparison condition (ARM 1): Length of contact (minutes) Number of contacts Content of treatment Duration of contact over time Provided information about treatment dose in the comparison condition (ARM 2):	NA	NA	NA	NA	NA	NA	
	2a	Length of contact (minutes) Number of contacts Content of treatment Duration of contact over time Method to ensure that dose is equivalent between conditions Method to ensure that dose is equivalent for participants within conditions	NA	NA	NA	NA	NA	NA	
	3	Specification of provider credentials that are needed	1	NA	NA	NA	NA	NA	
	4	Theoretical model upon which the intervention is based is clearly articulated: The active ingredients are specified and incorporated into the intervention Use of experts or protocol review group to determine whether the intervention protocol reflects the underlying theoretical model or clinical guidelines. Plan to ensure that the measures reflect the hypothesised theoretical constructs/mechanisms of action	1	NA	NA	NA	NA	NA	
	5	Potential confounders that limit the ability to make conclusions at the end of the trial are identified?	0	NA	NA	NA	NA	NA	
	6	Plan to address possible setbacks in implementation (i.e., back-up systems or providers)	0	NA	NA	NA	NA	NA	
	7	If more than one intervention is described, all described equally well	NA	NA	NA	NA	NA	NA	
**Training of** **Providers**: Involved ensuring that training was delivered by a competent provider, using a 2-day training workshop, developing a clear training plan that addressed bio-psycho-social aspects in CLBP, with a focus on the BPS model, patient education, physical activity delivery/exercises and evaluation of training delivered.	8	Description of how providers will be trained (manual of training procedures)	1	NA	NA	NA	NA	NA	Could be assessed using audiotaping or videotaping and having 2 or 3 independent assessors or voluntary researchers score the procedures using a checklist, and interrater reliability assessed [22, 65]. Also, a performance criterion could be developed based on the treatment components that physiotherapists would be trained on, and a 5-point Likert scale used to assess physiotherapists’ competence of the different treatment components, using role play [65].
	9	Standardisation of provider training (especially if multiple waves of training are needed for multiple groups of providers)	1	NA	NA	NA	NA	NA	
	10	Assessment of provider skill acquisition	1	NA	NA	NA	NA	NA	
	11	Assessment and monitoring of provider skill maintenance over time	1	NA	NA	NA	NA	NA	
	12	Characteristics being sought in a treatment provider are articulated a priori. Characteristics that should be avoided in a treatment provider are articulated *a* priori	1	NA	NA	NA	NA	NA	
	13	At the hiring stage, assessment of whether or not there is a good fit between the provider and the intervention (e.g., ensure that providers find the intervention acceptable, credible and potentially efficacious	1	NA	NA	NA	NA	NA	
	14	There is a training plan that takes into account trainees’ different education and experience and learning styles	0	NA	NA	NA	NA	NA	
**Treatment** **Delivery**: Involved assessing the delivery of patient education; supervised exercises and home exercises (5x weekly, 30 min daily) for 6weeks, by two physiotherapists. Adherence to the treatment manual content, duration and mode of delivery were assessed. Fidelity of treatment delivery was set at achieving adherence to delivering > 80% of the treatment components).	15	Method to ensure that the content of the intervention is delivered as specified	1	1	1	1	1	1	Could be assessed using audiotaping or videotaping and having 2 or 3 independent assessors or voluntary researchers score the procedures using a checklist, and interrater reliability assessed [22, 65]
	16	Method to ensure that the dose of the intervention is delivered as specified	1	1	1	1	1	1	
	17	Mechanism to assess if the provider actually adhered to the intervention plan or in the case of computer delivered interventions, method to assess participants’ contact with the information	1	1	1	1	1	1	
	18	Assessment of non-specific treatment effects	0	0	0	0	0	0	
	19	Used treatment manual	1	1	0	1	1	0	
	20	There is a plan for the assessment of whether or not the active ingredients were delivered	1	1	1	1	1	1	
	21	There is a plan for the assessment of whether or not proscribed components were delivered (e.g., components that are unnecessary or unhelpful)	1	1	1	1	1	1	
	22	There is a plan for how contamination between conditions will be prevented	NA	NA	NA	NA	NA	NA	
	23	There is an a priori specification of treatment fidelity (e.g., providers adhere to delivering > 80% of components)	1	1	1	1	1	1	
**Treatment** **Receipt**: Involved assessment of the understanding of the patient participants of the various components of the intervention. It involved assessing processes that were used to enhance patient participants understanding of the intervention being delivered (for example, explaining the patient education components with the examples patient participants could relate to).	24	There is an assessment of the degree to which participants understood the intervention	1	1	1	1	1	1	Could be assessed using audiotaping or videotaping and having 2 or 3 independent assessors or voluntary researchers score the procedures using a checklist, and interrater reliability assessed [22, 65]
	25	There is specification of strategies that will be used to improve participant comprehension of the intervention.	1	1	1	1	1	1	
	26	The participants’ ability to perform the intervention skills will be assessed during the intervention period.	1	1	1	1	1	1	
	27	A strategy will be used to improve subject performance of intervention skills during the intervention period	1	1	1	1	1	1	
	28	Multicultural factors considered in the development and delivery of the intervention (e.g., provided in native language; protocol is consistent with the values of the target group)	1	1	1	1	1	1	
**Treatment** **Enactment**: Involved assessment of the patients’ ability to apply the knowledge and skills learned around the intervention in real life contexts, using role-playing and checking understanding.	29	Participant performance of the intervention skills will be assessed in settings in which the intervention might be applied.	1	1	1	1	1	1	Could be assessed using audiotaping or videotaping and having 2 or 3 independent assessors or voluntary researchers score the procedures using a checklist, and interrater reliability assessed [22, 65]
	30	A strategy will be used to improve performance of the intervention skills in settings in which the intervention might be applied.	0	0	0	0	0	0	

Key: Has this been achieved: Present = 1, Absent but should be present = 0, NA = Not applicable

**Table 3:** Primary outcomes based on feasibility criteria

#	Outcome	Feasibility criteria	Result	Feasible/Acceptable	Comments
	Recruitment				
1	Screening for eligibility	Ability to screen all eligible patients	39 out of 39 screened	Yes	All 39 participants were successfully screened for eligibility by the principal investigator. Patients were excluded based on the eligibility criteria and their unwillingness to participate in the research. Reasons for non-eligibility; acute LBP (*n* = 1), post-surgery (*n* = 1) Not interested in participating (*n* = 1) Lumbar canal stenosis (*n* = 4) Unable to attend all sessions due to proximity (2)
2	Provision of information sheets and explanation of purpose for the research	Ability of the researcher and/or research assistant to deliver the information sheets and explain the purpose of the study to participants	30 out of 30 participants	Yes	All participants were given information sheets and the purpose of the research thoroughly explained to participants. Some participants (*n* = 6) needed explanation both in English and in the local language (Twi). Recommendation: Twi versions of the information sheets should be considered for participants who cannot read English.
3	Informed consent (consent rate)	Percentage of patient participants who consent to participate against the number of eligible patient participants	100%	Yes	All participants who were willing to participate signed the consent form to participate. The principal investigator explained aspects of the consent form where participants needed clarification. One eligible patient (among the 39 patients screened) declined to participate with reasons.
4	Recruitment rate	3 ≥ patient participants per week	5 patient participants per week (100% recruitment)	Yes	The referral rate of doctors at the family medicine directorate was similar to previous trends (average of 30 patients referred monthly) therefore facilitating the successful target of recruiting 30 patients with CLBP within 2 months. Actual recruitment spanned six weeks.
5	Patient allocation	Assignment of all patients (100%) to physiotherapist participants	100%	Yes	PT 1: 18 patients PT 2: 12 patients
	**Training**				
6	Training programme for physiotherapist participants	Whether physiotherapist participants can be successfully trained. Assessed based on positive feedback from physiotherapist participants. Reported on the training evaluation forms	Positive feedback achieved by the participating physiotherapists	Yes	Comments from the training evaluation form PT 1: • The setting was conducive for the training. • Yes • The delivery was clear, and the handouts too further explained what was delivered. • The entire session was useful and educative. • There was not such aspect in the training that were not relevant. PT 2: • The setting was a well-known and friendly environment, devoid of tension. A well organised lab session, where exercise demonstration and practice was feasible. • Objectives were stated before the training and were met after the training. • Method of delivery, that is; roundtable discussion was very effective, and questions asked clarified the purpose of study. • Discussions were more understanding. Exercise sessions was interesting. • Adding more categories of back patients.
	**Data completion**				
7	Baseline background information: Age, gender, religion, duration of LBP, date of onset, educational level, employment status, marital status	80% ≥ data completion	89.5%	Yes	One patient did not state the type of work (employment) they are involved in. One patient did not state her marital status. Only one patient indicated a specific date of onset of her LBP.
8	Rate of data completion: Outcome measures (Numeric rating scale Roland Morris Disability Questionnaire Quality of life/ Health Status – Euro-Qol EQ-5D-5 L Pain catastrophising scale General self-efficacy scale Tampa Scale of Kinesiophobia)	80% ≥ data completion	Pre-intervention = 99.2%	Yes	All participants were given questionnaires and missing data was less than 1% for pre-intervention, post-intervention, and 3-month follow-up outcome assessment. Some participants (*n* = 6; no formal education = 2, primary education = 4) sought explanation on some of the questions in the local language (Twi). Recommendation: Twi versions of the outcome measures should be considered for participants who cannot read English. This will necessitate validating outcome measures in Twi.
			Post- intervention = 99.8%	Yes	
			Three-month follow-up = 99.7%	Yes	
	**Retention**				
9	Retention rate:	80% ≥ retention of patient participants	90%	Yes	27 patients were retained in the study post-intervention. Reasons for dropout of three patients included patients relocated to a different region (*n* = 2) and unable to attend due to work (*n* = 1).
10	Dropout rate	≥ 20% dropout of patient participants	Post intervention = 10%	Yes	Three patient participants dropped out after the 1st, and 2nd (*n* = 2) sessions. Reasons included patients relocated to a different region (*n* = 2) and unable to attend due to work (*n* = 1).
			Three-month follow-up = 30%	No	A further 6 patients dropped out at three-month follow-up. There was no response after two reminders sent in a space of two weeks.
	**Treatment compliance**				
11	Adherence to outpatient BPS treatment sessions	80% ≥ patient participants’ adherence to treatment sessions	80.6%	Yes	The majority of patients (*n* = 20) completed at least 9 out of the 12 sessions within the six weeks study period. Session completion: 12 sessions = 4 11 sessions = 8 10 sessions = 4 9 sessions = 4 The rest completed 8 sessions (*n* = 1), 7 sessions (*n* = 5), 6 sessions (*n* = 1).
12	Adherence to home exercise programme	80% ≥ patient participants’ adherence to home programme	18.5%	No	The majority of patients (*n* = 22) did not adhere to the home programme. Only four (14.81%) patients adhered completely to the home programme. One patient completed 5 out of 5 days of the weekly home programme; however, only 20 min out of the 30 min threshold was achieved on average. The physiotherapist participants did not capture all data on patient’s home programme in the electronic folders. Patient reports from phone enquiry (by the research team; PI and voluntary research assistant) on reasons for non-adherence included: time constraints due to work and other family commitments Neighbourhoods not conducive for walking, and issues with timing and documentation
	**Fidelity and adverse events**				
13	Adverse events	Ability to capture data on adverse events by participating physiotherapists and whether any adverse events were captured	No adverse event data captured	Yes	It was feasible to collect the data. No adverse events were recorded, although 2 patients had to reschedule their sessions due to other medical conditions unrelated to their CLBP.
14	Fidelity of intervention	80% ≥ attainment of intervention fidelity	83.1%	Yes	Intervention fidelity was high. Although the treatment protocol was adhered to more than 80% of the time, physiotherapists deviated on some occasions. For example, in a situation where in patient education was delivered on a group basis instead of individual. Other factors which were not fully adhered to that reduced fidelity of the intervention included (based on the NIHBCC fidelity checklist): • Development of a strategy to improve performance of the intervention skills in settings in which the intervention might be applied. • Assessment of non-specific treatment effects. • Strict adherence to the use of the treatment manual provided.

### Secondary outcome measures

Data from the patient reported outcome measures was primarily analysed as an exploratory process to understand whether the data could be analysed. The results showed trends towards improvements for all clinical outcomes post-intervention compared to the pre-intervention/baseline data (Table 4).

**Table 4:** Results of clinical outcome measures, mean (SD)

Outcome	Baseline (Pre-test)	Post-test	Three months
NRS	7.4 (1.2)	2.8 (1.6)	3.6 (1.6)
RMDQ	15.9 (3.9)	2.7 (2.9)	3.3 (3.9)
EQ-5D-5 L	3.5 (0.5)	0.7 (0.5)	0.8 (0.5)
Health VAS	44.0 (10.7)	74.0 (10.6)	77.2 (9.5)
PCS	37.6 (7.9)	7.2 (7.9)	9.2 (5.1)
GSES	14.9 (2.8)	21.6 (6.0)	27.8 (6.4)
TSK	53.3 (5.7)	34.4 (6.1)	34.0 (5.2)

NRS; numeric rating scale, RMDQ, Roland Morris Disability Questionnaire, EQ-5D-5 L = EuroQol 5 dimensions, VAS; visual analogue scale, PCS; pain catastrophising scale; GSES, general self-efficacy scale; TSK = Tampa scale of kinesiophobia

### Qualitative interview results

#### Training programme

Overall, interviews lasted between 30 min and 70 min. Physiotherapist participants reported that the BPS intervention improved their understanding on the rationale for the study, with clarity/purpose.

*“It was well organised; we know where we are starting from*,* the next stage that we are going to*,* the expected outcome of it and all that.” (PT2)*.

*“The delivery was clear*,* and the handouts too further explained what was delivered” PT1*.

#### Recruitment and retention

##### Data completion

Participants reported that the allocated time for sessions was adequate, and were also clear on the content of the patient reported outcome measures.

*“Oh yes*,* it wasn’t short. One hour it’s OK. The duration was adequate for me.” (P3_55-Year-old female)*.

*“Oh yea I think they were all clear.” (P5_34-year-old female)*.

#### Treatment compliance

Patient compliance with treatment schedules was highlighted by participants, they reported no major issues.

*“….Anytime I come and go through my intervention*,* whatever I do that I don’t understand he is able to explain it*,* and he gave me a lot of encouragement*,* and you too when I met you even your smile gave me hope that at least I’m in the hands of good people*,* who can help me.” (P2_47-Year-old female)*.

However, there were instances related to external factors (for example, work issues and sickness) where non-compliance with treatment schedules resulted in patient drop-out.

*“There were just a few of them who would miss sessions and I realised most of them had tangible reasons to miss. Some of them too they fell sick were admitted and they couldn’t come for their appointment. But on the whole*,* I would say about 95% of them were compliant.” (PT1)*.

#### Adverse effects

Patient participants further reflected on the details of the BPS intervention regarding whether any adverse effects occurred. No adverse effects were reported with reports of the BPS intervention being safe also.

*“Yes*,* sir some were safe. There are some when I’m into it I don’t hear my pain*,* and there are some when its being introduced to me and when I start or through the process*,* I hear the language of the waist pain. It tells me the pain is there……I did not stop them sir. I continued having beliefs that with continuous and then time it would and then thank God through the process it escaped sir.” (P6_29-Year-old male)*.

*“Yes….yes I did*,* and throughout initially the exercises were painful and then with time it was OK. Normal if you know what you are doing and you know where you are going*,* you will be more comfortable” (P1_41-year-old female)*.

Overall, the patient participants’ reported that the BPS intervention was acceptable, they were satisfied and felt safe engaging with the intervention.

## Discussion

The results shows that it is feasible and acceptable to deliver the BPS intervention in a Ghanaian setting. The majority (*n* = 12) of feasibility criteria were achieved. The feasibility threshold for patient participants’ adherence to the home management and dropout rate at 3 months follow-up assessment were not achieved. These may necessitate further feasibility testing. In considering a larger clinical study for a BPS intervention, it will be important to ascertain whether participants will consent to be randomised. This will also necessitate further feasibility testing. Given the nature of the study, blinding participants to the intervention was not a requirement; nonetheless, the research team adhered to high ethical standards and robust processes throughout the operationalisation of the study. Furthermore, the improvement in important outcomes such as disability is consistent with a previous feasibility study in an LMIC [41]. Overall, the results demonstrate that the BPS intervention shows promise, and could be further explored in future studies.

### Processes (training, recruitment)

The use of a clinical gatekeeper facilitated a successful recruitment of physiotherapist and patient participants. The ability to recruit and retain HCPs in clinical studies is important to researchers [66]. Effective engagement of HCPs is enhanced where HCPs opinions are considered in developing the study [67]. The support from the gatekeeper was crucial; this is because, support from staff of a health facility, scepticism about the usefulness of a study, and limited research experience, are major barriers to HCPs engagement in research [68]. Regarding recruitment of patients, it is unclear what accounted for the high number of females. This evidence is however similar to global trends which shows that the prevalence of LBP is higher in females across all age groups [7]. It is noted that recruitment of participants for research studies is a challenging task [69]. Many clinical studies fail to recruit/enrol enough study participants [70, 71]. Therefore, achieving the recruitment target was a positive outcome.

### Study resource (compliance and compliance monitoring)

Patient compliance to treatment is a major challenge in clinical studies [72]. Although the research team put in strategies to reduce missed sessions (for example, reminders via text messages), missed sessions were record. The feasibility criteria regarding compliance with out-patient management sessions were met; however, the reasons for the missed sessions presents opportunities for further investigation. Regarding patient participants’ compliance with the home programme, monitoring was difficult by physiotherapists, and the majority of patients did not return their exercise diaries. The unsupervised nature of the home exercise programme appeared to facilitate the patients’ non-compliance. Previous reviews conclude that there is a lack of reliable and valid method of collecting patient-reported outcome measures for unsupervised exercise-based programmes [72, 73]. Therefore, the challenge of completing exercise diaries is not peculiar to this research and presents a challenge regarding the strategies to monitoring patients’ home programmes.

## Study management (fidelity, missing data/retention rate, attrition)

The high patient retention rate post intervention (90%) recorded in this study is similar to clinical studies from LIMICs [41, 74]. However, at three-month follow-up, a dropout rate of 30% was recorded, with a total of 9 patients being lost to follow-up. A high dropout rate at 3-month is not peculiar to the current study. Similar findings exist in studies from both HICs [31, 75] and LMICs [76]. In order to reduce patient dropout, it is important to incorporate strategies that will enhance patient participants’ engagement with the BPS intervention. These strategies may include regular reminders through phone calls, text messages, and emailing to aid patients in reporting outcome measures and assist researchers in collecting information on patient adherence [31]. The application of electronic/web-based approaches to data collection have however not offered a solution to outcome assessment [77]. This phenomenon therefore reveals the complex nature of seeking solutions to high patient dropout at follow-up assessment. It is important to note that a high patient dropout does not suggest that further clinical studies are not feasible [78, 79]. However, adjustments should be made for the rate of recruitment and calculation of the minimum sample size in such instances [31].

It was observed that 6 patient participants sought clarification of aspects of the outcome measures in the local language (Twi). This was probably attributable to a high illiteracy level since 2 out of the 6 patient participants had no formal education and the remaining 4 had primary education as their highest level of education. However, overall data completion rate was high for all data, which is similar to that of feasibility studies from both HICs [31] and LMICs [80]. Overall, the fidelity of the BPS intervention was high (83.1%). The high fidelity of the BPS intervention recorded is comparable to the fidelity achieved in a UK-based feasibility study [31]. A systematic review (22 studies) by Toomey et al., [81], investigating the fidelity of physiotherapist-led interventions for the management of patients with CLBP showed that the majority of studies (*n* = 21) had very low fidelity scores (mean score of 36%). Similar feasibility studies from a LMIC (Nigeria) did not assess and/or report the fidelity of their interventions [41, 74].

### Strengths and limitations of this study

This study is the first to investigate the feasibility of a BPS patient education and exercise intervention in a Ghanaian out-patient hospital setting. This is a novelty regarding healthcare research in Ghana. This study is also the first to test the acceptability of training physiotherapists to deliver a BPS intervention in a Ghanaian hospital setting. Obtaining high-fidelity scores in a feasibility study is important for future clinical studies because it demonstrates the potential to replicate the interventions protocol as planned [82]. The application of qualitative and quantitative methods in this study strengthens the validity of the findings [83]. Methodologically, the strengths include the application of robust data collection processes, triangulation of quantitative and qualitative data for corroboration [84], and a clear audit trail of the qualitative study. However, the application of a quasi-experimental method may be criticized as not being highly robust. Although quasi-experimental studies are not as robust as RCTs [85], applying it was a first step to understand the feasibility of delivering the BPS intervention in Ghana. This study was limited due to non-inclusion of a control group, hence the feasibility/acceptability of randomly allocating patients to control groups is unknown. Furthermore, the non-random allocation of patient participants could have resulted in a selection bias.

## Conclusion

This study has offered new knowledge into the feasibility of delivering a BPS intervention programme for patients with CLBP in Ghana. The results have established the achievement of many feasibility/assessment criteria. Overall, the outcome of this study demonstrates promise for the delivery of the BPS intervention and serves as an important platform for the development of further knowledge in Ghana.

## Electronic supplementary material

Below is the link to the electronic supplementary material.

Supplementary Material 1

Supplementary Material 2

Supplementary Material 3

Supplementary Material 4

Supplementary Material 5

Supplementary Material 6

## Data Availability

The data that support the findings of this study are available from the University of Nottingham, Faculty of Medicine and Health Sciences Research Ethics Committee, but restrictions apply to the availability of these data, which were used under license for the current study, and so are not publicly available. Data are however available from the authors upon reasonable request and with permission of University of Nottingham, Faculty of Medicine and Health Sciences Research Ethics Committee.
