# Supplementary material for: A physiotherapist-led biopsychosocial education and exercise programme for patients with chronic low back pain in Ghana: a mixed-methods feasibility study
Source: BMC Musculoskelet Disord. 2024 Dec 18;25:1014. doi: 10.1186/s12891-024-08118-1 (PMC11654333; doi:10.1186/s12891-024-08118-1)
Supplement: Supplementary file 1 — Supplementary Material 1 [file 12891_2024_8118_MOESM1_ESM.docx]

**Supplement 1: Participants’ eligibility criteria**

| **Inclusion criteria-Patients** | **Justification** |
| --- | --- |
| Male and female adult patients 18 years and over | This allowed for feasibility and secondary outcomes to be assessed across all genders and the aetiology/factors involved with adolescent/childhood conditions may significantly differ from the adult population. Therefore, this study focussed on the adult population. |
| Presence of CLBP, that is, low back pain lasting 3 months or more who have been listed on the physiotherapy waiting list at the study site. | We defined LBP was defined as tension, pain or stiffness in the area located above the gluteal folds and below the costal margins with or without referred leg pain [29,30]. Recruitment of patients was restricted to patients who were listed to receive physiotherapy at only the study site-KATH. |
| Patients with clinical diagnosis of non-specific chronic low back pain | We defined non-specific CLBP as LBP lasting more than 3 months without any readily identifiable cause or not associated with any serious pathology such as malignancy, neurological conditions, infection, fractures, spondyloarthropathy, malignancy, abdominal aortic aneurysm [30,31]. |
| Willingness to participate | Participation in this study was entirely voluntary. |
|  |  |
| **Inclusion Criteria-Physiotherapists** | **Justification** |
| Licensed Male and female physiotherapists actively involved with the management of CLBP at the study site- KATH | This allowed for inclusion of qualified physiotherapists, and restricted recruitment to the study setting. |
| Physiotherapists with varied years of working experience, ranks and qualification. | This allowed for inclusion of physiotherapists with varied backgrounds. This recruitment approach ensured access to a broad variety of physiotherapists by the research team. |
|  |  |
| **Exclusion criteria-Patients** | **Justification** |
| Evidence of severe pathology of the spine/specific causes of CLBP (for example, fracture, infections, stenosis of the spine, inflammatory diseases, tumours)^30,31^. | The study was restricted to patients presenting with non-specific CLBP. |
| Pregnant women. | Pregnancy may be a specific underlying cause of CLBP [31]. |
| Presence of severe psychological illness or unable to understand and/or sign consent forms. These may predispose to medico-legal issues. | The research was conducted within acceptable ethical and medico-legal considerations. |
|  |  |
| **Exclusion criteria-Physiotherapists** | **Justification** |
| Physiotherapy assistants and healthcare assistants | Physiotherapists directed patents’ treatments and made all the decisions regarding patient care in the study setting. |
| Unwillingness to participate | Participation in the study was entirely voluntary |
