# Supplementary material for: A physiotherapist-led biopsychosocial education and exercise programme for patients with chronic low back pain in Ghana: a mixed-methods feasibility study
Source: BMC Musculoskelet Disord. 2024 Dec 18;25:1014. doi: 10.1186/s12891-024-08118-1 (PMC11654333; doi:10.1186/s12891-024-08118-1)
Supplement: Supplementary file 2 — Supplementary Material 2 [file 12891_2024_8118_MOESM2_ESM.docx]

| **Supplement 3: BPS intervention protocol** | | | | |
| --- | --- | --- | --- | --- |
| **Patient education** | | | | |
| **Step** | | **Topic/focus** | **Goal** | **Activity/message delivered** |
| Week 1 | | Interactive session/  discussions/questions | To establish a good rapport and relationship  To set goals  To explore participants beliefs about low back pain (LBP) | Treatment rationale and expected goals.  Importance of realistic goals setting or action plans.  Participants will be allowed to tell their story/experience about LBP.  Unhelpful beliefs or information obtained in this step will be addressed in subsequent steps |
|  | | Meaning of LBP | To promote an understanding of the meaning of LBP | Definition of LBP, nonspecific LBP versus specific LBP, acute versus chronic or persistent LBP (CLBP).  Brief epidemiology of LBP.  Disability related to LBP and the burden of LBP. |
|  | | Common facts about CLBP | To understand the common facts/myths about CLBP | LBP is common, may not be due to any disease or pathology, and reoccurrence is common but still does not mean the presence of a disease or pathology.  Extended bed rest does not help and may actually prolong pain and leads to more disability. |
|  | | | | |
| Week 2 | | Common beliefs about LBP | To reshape false or unhelpful  beliefs about LBP | Beliefs of having a serious injury, fear of movement due to pain or damage, beliefs about work or physical activity and pain are linked, excessive attention on pain, total bed rest or inactivity, and over-reliance on medications will be addressed as unhelpful.  Beliefs about LBP and infertility or impotence are linked, and other beliefs learned from participants that are un related to LBP will all be addressed.  False beliefs about pain can prolong the pain experience.  Avoiding movement and or activity due to fear of pain has negative consequences and can lead to pain persistence and loss of function.  Total bed rest leads to stiff joints, weak muscles and bones, decline physical fitness, and reduced function.  Pain medications can adverse effects in the long-term. |
|  | | Basic anatomy | To promote an understanding of the back (spine) as one of the strongest structures in the body | The spine is made of solid bony blocks joined by discs to give it strength and flexibility.  It is reinforced by strong ligaments and surrounded by large and powerful muscles which protect it.  Due to the inherent strength of the spine, it is not easily damaged. |
|  | | Pain causation | To promote a better understanding of the cause of pain | Feeling pain does not necessarily mean tissue injury or damage as pain and image diagnostics (e.g., X-ray) correlates poorly. Scans are more useful for specific LBP such as fractures.  In most people, it is difficult to identify the exact source of the problem. Though it may be frustrating, it is good news in another way you do not have any serious injury or damage in your spine.  A simple back strain does not result in any permanent damage.  Permanent serious back injuries are usually caused by high-energy trauma.  Most people with LBP do not have any damage in their spine.  Many people have disc bulge or degeneration but have no symptoms of LBP.  Presence of such changes may not be predictive of future pain.  Even though some people with back pain have slipped disc, it usually gets better by its self and very few cases ever require surgery. |
|  | | | | |
| Week 3 | | Basics of pain physiology | To promote basic knowledge about pain mechanism and common factors influencing it | Meaning of pain.  Basic nociceptive pathways: nociceptors, spinal cord, and brain.  Why do we get pain? Pain is an alarm (warning), meant to protect and motivate to create an action.  Pain gate: gate that controls flow of signals (pain messages) between the body and the brain.  Examples of factors that aggravate pain includes believing that hurt means harm, fear of movement due to pain, pain catastrophizing, depression, anxiety, stress, tension, focusing on pain, sadness, lack of social support, and anger. These factors also play a key role in leading to CLBP.  Examples of factors that close the gate include happiness/laughter, physical activity, exercises/stretching.  Knowledge of these factors is important in reducing the factors that predispose a patient to chronicity.  Since we know what influences our pain, we can understand that pain is not necessarily due to injury or damage in the spine. It can be felt with no changes to the body structure. |
|  | | Return to normal activities  and stay active | To encourage the early return to normal activities and the  importance of remaining  active despite in pain | Make an early return to normal or vocational activities as tolerated without thinking that activities of daily living are harmful.  Since the muscles, ligaments or joints of the spine helps you get moving and most pain are felt from these structures, when you stop moving, these structures slowly lose their function leading to disability.  To get your back working properly, you must move (physical activity).  It is safe to stay active and the sooner you get active, the sooner your back will feel better.  Avoid unaccustomed or extended bed rest when there seems to be serious pain or overdo activities when there seems to be less pain. This is crucial in dealing with an acute attack, enables you to recover better, and can prevent recurrence.  Physical activity even with pain is unlikely to further damage your back when the pain is a result of NSLBP. |
|  | | Pain coping and pacing | To promote better active coping through adopting safe and effective pacing | Monitor your symptoms and identify the likely contributing factors to your pain exacerbations or amelioration. Safe pacing (e.g., alternating activity with rest, slowing down when performing tasks), especially during flare-ups, is useful.  Modify your activity, adopt positive attitudes and engage in a variety of meaningful activities despite being in pain. Do not let your back take over your life.  Performance of more natural spinal movements in less pain is essential to good pacing.  Pacing for common activities participants engage in will be discussed. |
|  | | | | |
| Week 4 | | Self-management | To promote active  self-management strategies  and reduce over-reliance on  formal health care utilization | Effective self-care strategies are important in coping with pain and enhancing recovery.  Self-care options including the use of common pain relievers (only prescribed by the physician), heat and cold packs, massage (with topical pain creams), stretching exercises, aerobic exercises (e.g., walking activities), and relaxation techniques (e.g., listening to music, dancing, attending social events) will be advised/taught.  Do not rely on single treatment; a combination of approaches will likely have the greatest benefit. |
|  | | Postural hygiene | To promote healthy postural habit at home or at work as means of reducing the risk of temporary pain episodes | Postural modification is important to reduce risk of temporary pain episodes from physical overload or prolonged static activities. No clear correlation between posture and pain.  Postural modifications for common daily tasks/activities such as standing, sitting, bending, and lifting will be taught as a means of reducing back muscle tension to ease pain.  Special considerations will be given to some common practices such as heavy manual lifting. For example, the use of modifiable tools and other means of carrying items will advised. |
|  | | | | |
| Week 5 | | Increasing activity level | To promote the importance of  improving physical activity levels | Gradually increase physical activity levels that are tolerable, comfortable and safe.  Moderate physical activities (based on the American College of Sports Medicine’s position statement) may include over ground brisk walks, cycling, washing clothes, and swimming.  Plan your days and have some exercise every day. You can try walking or cycling instead of going by bus, or motorcycle.  Regular activity develops muscles, gives stronger bones, release natural chemicals that reduce pain, promote fitness and sense of well-being.  Monitor your own functional progress and do more progressively. |
|  | | Lifestyle modification | To promote a healthy lifestyle and reduce risk of additional problems | Physical inactivity, sedentary lifestyle, obesity, smoking, sleeping less, and stress can have negative direct or indirect impact on your back and overall health.  Adopt a healthy lifestyle, have adequate sleep (at least 7 hours per day), reduce or stop smoking, avoid physical and mental stress, and eat healthy (balanced diet). Also, maintain good social participation. |
|  | | | |  |
| Week 6 | | Warning signs of LBP and  what to do | To promote an understanding of warning signs (red flags) of LBP and the importance of a hospital visit when necessary | In case of signs such as weight loss, night sweating, leg weakness, sensory disturbances (pins and needles) around the buttocks, anus, genital area or inner surfaces of the thighs and difficulty in passing or controlling urine/bowel, consult a physician immediately.  These symptoms, however, are rare thus you do not need to worry about them. |
|  | | Review of discussions and  applications | To evaluate understanding and application of information/ programme learned | Previous concepts learned will be reviewed. Their application will be discussed. Areas of doubt or requiring additional explanation will be further discussed. |
|  | | | | |
| **Motor control exercises** | | | | |
| **Stage/progression** | **Exercise** | | | |
| Stage 1  (1st–3rd  sessions) | 1. Abdominal drawing-in manoeuvre (ADIM) in supine  2. ADIM in quadruped  3. ADIM in sitting  4. ADIM in standing | | | |
|  |  | | | |
| Stage 2  (4th–9th  sessions) | 5. ADIM in supine with heel slide (each leg)  6. ADIM in supine with leg lift (each leg)  7. ADIM in supine with bridging (two legs)  8. ADIM in supine with single-leg bridge  9. Supine ADIM with curl-up (elbows on the table)  10. Supine ADIM with curl-up (hands over the forehead)  11. ADIM in horizontal side support with knees bent  12. ADIM in horizontal side support with knees straight  13. Side-lying horizontal side support with ADIM  14. ADIM in quadruped with arm raise  15. ADIM in quadruped with leg raise  16. ADIM in quadruped with alternate arm and leg raise | | | |
|  |  | | | |
| Stage 3  (10th–12th  sessions) | 17. Rolling from side to side with ADIM  18. Sit-stand transfer with ADIM  19. Wall squatting with ADIM  20. Walking with ADIM (10 min) | | | |
|  | | | | |
| **Stretching exercises (to be included in sessions)** | | | | |
| **Exercise** | **Description** | | | |
| Double knees to chest stretch | In a supine lying position with the knees bent and feet flat on the couch, interlock fingers just under the knees and gently pull towards the chest to the maximum tolerable level. | | | |
| Piriformis stretch | In a supine lying position with the knees bent and feet flat on the couch, the ankle of one leg crossed over the opposite hip crease. Interlock fingers just under the other knee and gently pull towards the chest until a comfortable stretch is felt. Switch sides. | | | |
| Hamstring stretch | In a supine position, while keeping the knee and hip extended, extend the knee progressively with the foot moving towards the ceiling until a stretch is felt in the posterior aspect of the knee/thigh. Switch sides. | | | |
| Trunk rotation | In a supine lying position, cross the right foot over the left knee, using the left hand gently pull the right knee towards the floor while twisting the spine to the right and keeping the right arm and shoulder straight out on the floor. Switch sides. | | | |
| Erector spinae stretch | While sitting on the heels, bend the trunk with the abdomen resting on the front of the thighs while stretching arms forward. | | | |
| Hip adductor stretch | While sitting upright on the floor with soles of the feet together and the heels closes to the body, gently press down the knees with the hands until a comfortable a stretch is felt in the inner thigh region. | | | |
| Triceps surae stretch | In a standing position with both feet at a distance of 2 steps from a wall and both hands on a wall for balance, one leg is stretched in its place while taking a step forward with the other leg. Switch sides. | | | |
| Trunk extension stretch | In a standing position with the feet shoulders-width apart, place the hands on the pelvis and slowly bend the back backward as far as possible until a comfortable stretch is felt. | | | |
| Adapted from: Ibrahim et al.,[43] | | | | |
