# Supplementary material for: A physiotherapist-led biopsychosocial education and exercise programme for patients with chronic low back pain in Ghana: a mixed-methods feasibility study
Source: BMC Musculoskelet Disord. 2024 Dec 18;25:1014. doi: 10.1186/s12891-024-08118-1 (PMC11654333; doi:10.1186/s12891-024-08118-1)
Supplement: Supplementary file 3 — Supplementary Material 3 [file 12891_2024_8118_MOESM3_ESM.docx]

**Supplement 6: Secondary outcome measures applied in this study**

| **#** | **Assessment** | **Outcome measure** | **Comment(s)/Rationale** |
| --- | --- | --- | --- |
| 1 | Kinesiophobia | Tampa scale of kinesiophobia (TSK) | Kinesophobia/fear avoidance behaviours have been associated with increased pain sensitivity and decreased quality of life in patients with CLBP; and is an important psychological predictor of CLBP disability [48]. The 17-point TSK was used to measure avoidance behaviours due to fear (Kinesophobia) [49]; TSK has good internal consistency and construct validity [50]. |
| 2 | Quality of life | Generic health outcome Euro-QOL (EQ-5D-5L) | Quality of life is defined as an individual’s perception about themselves, in relation to life goals, expectations and standards [51]. Quality of life provides an important and holistic measure of bio-psycho-social aspects that may affect the health outcomes of CLBP patients. The generic health outcome Euro-QOL (EQ-5D-5L) was used to assess the general quality of life of patient participants with regards to selfcare, pain and discomfort, anxiety and depression, mobility and usual activities; EQ-5D-5L has been extensively applied in both research and clinical settings internationally [52], and has good construct validity [53]. |
| 3 | Catastrophising | Pain catastrophising scale (PCS) | Pain catastrophising refers to heightened negative interpretation of pain, leading to exaggeration of actual pain symptoms [54]. Catastrophising has been found to be a psychological and prognostic factor in CLBP, predicting outcomes such as disability, pain intensity and return to work [55]. The PCS was used to assess patient participants’ level of catastrophising; the 13-point PCS scale details participants feelings and thoughts about their painful experience [56]. |
| 4 | Disability | Roland Morris Disability Questionnaire (RMDQ) | Disability is one of the main challenges associated with CLBP. CLBP has been described as a leading cause of disability globally [2]. CLBP related disability is associated with decreased quality of life, increased pain and psychological distress. The RMDQ was used in measuring patient participants’ function; RMDQ has good construct validity [57], and test-retest reliability [58]. |
| 5 | Self-efficacy | General Self-efficacy Scale (GSES) | According to Bandura [59], self-efficacy refers to belief in oneself to be able to overcome a challenging situation (e.g., back pain). Self-efficacy is an important psychological factor that has a positive effect on CLBP outcomes. It has been found to be negatively associated with pain, disability and psychological distress [60]. The GSES was used to assess patient participants’ self-efficacy (a strong predictor of disability amongst persons with musculoskeletal pain) [61]. The GSES was used to investigate patient participants’ capacity to manage unanticipated circumstances, solve problems, complete targets, and ability to cope [62]. The GSES has been extensively applied in clinical and research settings, and has strong reliability and validity [63]. |
| 6 | Pain intensity | Numeric rating Scale | Pain is one of the commonest symptoms for which patients presenting with CLBP report to hospitals. Chronic pain is known to be mediated by other psychosocial and biomedical factors; and therefore, considered a biopsychosocial phenomenon, due to the multifaceted contributors to pain [64]. The NRS was utilised to measure patient participants’ pain intensity; the NRS has good validity in relation to responsiveness [65]. |
