# Supplementary material for: A physiotherapist-led biopsychosocial education and exercise programme for patients with chronic low back pain in Ghana: a mixed-methods feasibility study
Source: BMC Musculoskelet Disord. 2024 Dec 18;25:1014. doi: 10.1186/s12891-024-08118-1 (PMC11654333; doi:10.1186/s12891-024-08118-1)
Supplement: Supplementary file 4 — Supplementary Material 4 [file 12891_2024_8118_MOESM4_ESM.docx]

**Supplement 5: Primary outcome measures: Study enrolment, assessment, intervention, and feasibility thresholds applied in the study**

| **#** | **Assessment criteria/Operational definition/ Timepoint** | **Enrolment** | **Pre-test assessment** | **Post-test assessment** | **Three-month follow-up** | **Qualitative Interviews** | **Feasibility threshold/Acceptability criteria** |
| --- | --- | --- | --- | --- | --- | --- | --- |
|  | **Recruitment:** Refers to the number of physiotherapists, and patient participants recruited weekly and allocated to participant physiotherapists. These were based on the five components below. | **√** |  |  |  |  |  |
| 1 | Screening for eligibility | **√** |  |  |  |  | Screening of all potential patients on the doctor’s referral list in the physiotherapy department of KATH for eligibility |
| 2 | Provision of information sheets and explanation of purpose for the research | **√** |  |  |  |  | Provision of information sheets and explaining the purpose of the study to all eligible patients on the doctor’s referral list in the physiotherapy department of KATH |
| 3 | Informed consent (consent rate) | **√** |  |  |  |  | 80% ≥ of eligible patients consenting to participate in the study |
| 4 | Recruitment rate (Physiotherapists and patients) | **√** |  |  |  |  | 3 ≥ participants per week over two months for patients.  2 ≥ physiotherapists |
| 5 | Patient allocation |  | **√** |  |  |  | Assignment of all consenting patients to physiotherapist participants |
|  |  |  |  |  |  |  |  |
|  | **Training:** Refers to the evaluation of the training delivered to physiotherapists, using developed evaluation forms. Forms were completed by the participant physiotherapists. This was done to assess the acceptability of the training programme delivered to physiotherapists. |  |  |  |  |  |  |
| 6 | Training programme for physiotherapist participants | **√** |  |  |  |  | Whether physiotherapist participants can be successfully trained. Assessed based on positive feedback or otherwise from physiotherapist participants. Reported on the training evaluation forms |
|  |  |  |  |  |  |  |  |
|  | **Data completion Rate:** This refers to the rate of completion (percentage) of the baseline characteristics and PROMs/secondary outcome measures by the patient participants. |  |  |  |  |  |  |
| 7 | Baseline background information:  Age, gender, religion, duration of LBP, date of onset, educational level, employment status, marital status |  | **√** |  |  |  | 80% ≥ data completion |
| 8 | Rate of data completion at post-intervention and 3-months follow-up:  Outcome measures (Numeric rating scale, Roland Morris Disability Questionnaire,  Health Status – Euro-Qol EQ-5D-5L, Pain catastrophising scale, General self-efficacy scale, Tampa Scale of Kinesiophobia) |  |  | **√** | **√** |  | 80% ≥ data completion |
|  |  |  |  |  |  |  |  |
|  | **Retention:** This was assessed via two components- retention rate & dropout rate. Retention rate refers to the percentage of patient participants that were retained at the assessment periods (i.e., post-intervention and 3-months follow-up). Drop-out rate refers to the percentage of patient participants that were lost at the assessment periods. |  |  |  |  |  |  |
| 9 | Retention rate |  |  | **√** |  |  | 80% ≥ of participants accounted for (based on the number of outcome forms returned) post intervention |
| 10 | Dropout rate |  |  | **√** |  |  | Maximum dropout of 20% of participants post intervention and at 3-month follow-up |
|  |  |  |  |  |  |  |  |
|  | **Treatment compliance rate:** This refers to percentage of patient participants’ who adhered to:  -outpatient physiotherapy sessions (assessed through attendance/duration of treatment)  -home programme (assessed using patients’ exercise diary) |  |  |  |  |  |  |
| 11  12 | Treatment compliance:  Adherence to outpatient treatment sessions  Adherence to home programme |  | **√**  **√** | **√**  **√** |  |  | 80% ≥ of participants completing scheduled management sessions.  80% ≥ of participants completing recommended home programme |
|  |  |  |  |  |  |  |  |
|  | **Fidelity and adverse events** |  |  |  |  |  |  |
| 13 | Adverse events: Defined as the ability of participant physiotherapists to identify and document adverse events. |  | **√** | **√** |  |  | Ability to capture data on adverse events by participating physiotherapists and whether any adverse events were captured |
| 14 | Fidelity of intervention: Defined as the procedures applied to observe and improve the validity and reliability of behavioural interventions (Borrelli et al., 2005). The NIHBCC checklist was used to measure fidelity. Five components (Study design, training, delivery, receipt, and enactment) were assessed by the PI/voluntary research assistant. Study design was assessed by considering both physiotherapist and patient aspects in the study. Training and delivery were assessed by observing participant physiotherapists, while receipt and enactment were assessed by observing patient participants. |  | **√** | **√** |  |  | Intervention meeting 80% ≥ of National Institute of Health Behaviour Change Consortium checklist for measuring treatment fidelity |
|  |  |  |  |  |  |  |  |
|  | **Qualitative data** |  |  |  |  |  |  |
|  | Assessment of acceptability, utility appropriateness of outcome measures from patient and physiotherapist participants |  |  |  |  | **√** | Positive response from qualitative interviews |
|  | Identification of potential barriers and facilitators for a potential delivery of the BPS approach in clinical practice from patient and physiotherapist participants |  |  |  |  | **√** | Response from qualitative interviews |
|  | Assessment of the extent to which management expectations were met from patient participants |  |  |  |  | **√** | Positive response from qualitative interviews |
