# Supplementary material for: A physiotherapist-led biopsychosocial education and exercise programme for patients with chronic low back pain in Ghana: a mixed-methods feasibility study
Source: BMC Musculoskelet Disord. 2024 Dec 18;25:1014. doi: 10.1186/s12891-024-08118-1 (PMC11654333; doi:10.1186/s12891-024-08118-1)
Supplement: Supplementary file 5 — Supplementary Material 5 [file 12891_2024_8118_MOESM5_ESM.docx]

| **Supplement 2: BPS training programme outline – Physiotherapists will be given copies of a training pack (containing the BPS intervention protocol and resources on the BPS model).** | | |
| --- | --- | --- |
| **Day 1** | | |
| **Activity/Objective/Topic** | | |
|  | Interactive session/objectives for the day/discussions/questions | |
|  | The Biopsychosocial model – Inception, perspectives in healthcare, and application in clinical practice for the management of patients with musculoskeletal pain | |
|  | Clinical course of LBP and the prognostic factors that predispose patients to chronicity | |
|  | Effectiveness of the BPS approach in managing patients with CLBP | |
|  | Rationale for the selected intervention | |
|  | | |
| **Training on BPS intervention protocol (Patient education component)** | | |
|  | Meaning of LBP - To promote an understanding of the meaning of LBP | |
|  | Common facts about CLBP - To understand the common facts/myths about CLBP | |
|  | Common beliefs about LBP - To reshape false or unhelpful beliefs about LBP | |
|  | Basic anatomy - To promote an understanding of the back (spine) as one of the strongest structures in the body | |
|  | Pain causation - To promote a better understanding of the cause of pain | |
|  | Basics of pain physiology - To promote basic knowledge about pain mechanism and common factors influencing it | |
|  | Return to normal activities and stay active - To encourage the early return to normal activities and the importance of remaining active despite in pain | |
|  | Pain coping and pacing - To promote better active coping through adopting safe and effective pacing | |
|  | Self-management - To promote active self-management strategies and reduce over-reliance on formal health care utilization | |
|  | Postural hygiene - To promote healthy postural habit at home or at work as means of reducing the risk of temporary pain episodes | |
|  | Increasing activity level - To promote the importance of improving physical activity levels | |
|  | Lifestyle modification - To promote a healthy lifestyle and reduce risk of additional problems | |
|  | Warning signs of LBP and what to do - To promote an understanding of warning signs (red flags) of LBP and the importance of a hospital visit when necessary | |
|  | Review of discussions and applications - To evaluate understanding and application of information/ programme learned | |
|  | | |
| **Day 2** | | |
| **Activity/Objective/Topic** | | |
|  | Interactive session/objectives for the day/discussions/questions | |
|  | Evidence for exercise/physical activity for the management of patients with CLBP | |
|  | Rationale for the selected intervention | |
|  | | |
| **Training on BPS intervention protocol (Exercise component)** | | |
| **Motor control exercises** | | |
|  | Stage 1  (1st–3rd  sessions) | 1. Abdominal drawing-in manoeuvre (ADIM) in supine  2. ADIM in quadruped  3. ADIM in sitting  4. ADIM in standing |
|  | Stage 2  (4th–9th  sessions) | 5. ADIM in supine with heel slide (each leg)  6. ADIM in supine with leg lift (each leg)  7. ADIM in supine with bridging (two legs)  8. ADIM in supine with single-leg bridge  9. Supine ADIM with curl-up (elbows on the table)  10. Supine ADIM with curl-up (hands over the forehead)  11. ADIM in horizontal side support with knees bent  12. ADIM in horizontal side support with knees straight  13. Side-lying horizontal side support with ADIM  14. ADIM in quadruped with arm raise  15. ADIM in quadruped with leg raise  16. ADIM in quadruped with alternate arm and leg raise |
|  | Stage 3  (10th–12th  sessions) | 17. Rolling from side to side with ADIM  18. Sit-stand transfer with ADIM  19. Wall squatting with ADIM  20. Walking with ADIM (10 min) |
|  | | |
| **Stretching exercises** | | |
|  | Double knees to chest stretch | In a supine lying position with the knees bent and feet flat on the couch, interlock fingers just under the knees and gently pull towards the chest to the maximum tolerable level. |
|  | Piriformis stretch | In a supine lying position with the knees bent and feet flat on the couch, the ankle of one leg crossed over the opposite hip crease. Interlock fingers just under the other knee and gently pull towards the chest until a comfortable stretch is felt. Switch sides. |
|  | Hamstring stretch | In a supine position, while keeping the knee and hip extended, extend the knee progressively with the foot moving towards the ceiling until a stretch is felt in the posterior aspect of the knee/thigh. Switch sides. |
|  | Trunk rotation | In a supine lying position, cross the right foot over the left knee, using the left hand gently pull the right knee towards the floor while twisting the spine to the right and keeping the right arm and shoulder straight out on the floor. Switch sides. |
|  | Erector spinae stretch | While sitting on the heels, bend the trunk with the abdomen resting on the front of the thighs while stretching arms forward. |
|  | Hip adductor stretch | While sitting upright on the floor with soles of the feet together and the heels closes to the body, gently press down the knees with the hands until a comfortable a stretch is felt in the inner thighs region. |
|  | Triceps surae stretch | In a standing position with both feet at a distance of 2 steps from a wall and both hands on a wall for balance, one leg is stretched in its place while taking a step forward with the other leg. Switch sides. |
|  | Trunk extension stretch | In a standing position with the feet shoulders-width apart, place the hands on the pelvis and slowly bend the back backward as far as possible until a comfortable stretch is felt. |
|  | | |
| **Resources**   1. The Back Book: Burton et al.,[34] 2. The Back revolution: Waddell,[35] 3. International practice guidelines: Wong et al.,[36] 4. Global Spine Care Initiative on the management of neck pain and NSLBP in developing countries: Chou et al.,[37] 5. The pain toolkit (short version): Moore and Cole [38] 6. Lorimer Moseley - Why Things Hurt: <https://www.youtube.com/watch?v=gwd-wLdIHjs> | | |
