# Supplementary material for: A physiotherapist-led biopsychosocial education and exercise programme for patients with chronic low back pain in Ghana: a mixed-methods feasibility study
Source: BMC Musculoskelet Disord. 2024 Dec 18;25:1014. doi: 10.1186/s12891-024-08118-1 (PMC11654333; doi:10.1186/s12891-024-08118-1)
Supplement: Supplementary file 6 — Supplementary Material 6 [file 12891_2024_8118_MOESM6_ESM.docx]

| **Supplement 4: Mapping the components of the intervention to the definition of a BPS intervention** | | |
| --- | --- | --- |
| **BPS definition** | **Intervention protocol of the study** | **Comments** |
| Physical | **Exercise component**   - Abdominal drawing-in manoeuvre exercise - Stretching exercises - Moderate intensity aerobic exercises | The rationale for the exercises was to promote physical activity and stimulate patients’ self-management of their CLBP |
| Psychosocial | **Patient education component**   - Promoting an understanding of the meaning of LBP. - Understanding the common facts/myths about CLBP and reshaping false or unhelpful beliefs about LBP. - Promoting an understanding of the back (spine) as one of the strongest structures in the body. - Promote a better understanding of the cause of pain. - Promoting basic knowledge about pain mechanism and common factors influencing it. - Encouraging the early return to normal activities and the importance of remaining active despite the presence of pain - Promoting better active coping through adopting safe and effective pacing. - Promoting active self-management strategies - Promoting healthy postural habit at home or at work as means of reducing the risk of temporary pain episodes. - Promoting the importance of improving physical activity levels. - Promoting a healthy lifestyle and reduce risk of additional problems. - Promoting an understanding of warning signs (red flags) of LBP and the importance of a hospital visit when necessary. | The rationale for the exercises was to:   - Promote patients’ knowledge about pain. - Promote patients’ awareness of the cognitive aspects of pain - Promote patients’ physical activity - Promote patients’ self-management |
